# Supplementary material for: Exploring determinants of formation of cognitive anchors from altruistic messages: A fuzzy DEMATEL approach
Source: PLoS One. 2023 Nov 6;18(11):e0293841. doi: 10.1371/journal.pone.0293841 (PMC10627445; doi:10.1371/journal.pone.0293841)
Supplement: S1 File — (DOCX) [file pone.0293841.s001.docx]

**A1 FILE: SUPPORTING INFORMATION**

**A. Step by step execution of the F-DEMATEL procedure**

**Step 1: Determining of influencing factors in the system**

Table A1.1 below indicates the factors that were used in the study after the literature survey and the three M-Delphi rounds.

**Table A1.1. The 12 Factors Used in the Study**

| **Factor** | **Description** |
| --- | --- |
| Naming | The assigning of names or labels to a message. |
| Emotional anchoring | The use of language that appeals to people’s feelings. |
| Thematic anchoring | The use of themes that are well-established and known by the audience. |
| Use of antinomies | The use of contradictions and paradoxes to push for more enquiry and understanding by the audience. |
| Use of metaphors | The use of symbolic language and imagery in the communication process. |
| Understanding the cognitive ability of the audience | Considering the audience’s thought processes, patterns and psychological tendencies when they process information. |
| Repetition | Presenting the same message to the audience several times. |
| Crafting engaging information/messages | Creating compelling and persuasive communication to ensure that people accept the information. |
| Timing | The strategic placement and delivery of a message to ensure that the audience is exposed to the message at the right time. |
| Cultural consideration | Taking into account the societal norms, values and beliefs of the audience when crafting and delivering messages. |
| Consistency | Ensuring that the core meaning of the message is the same over time. |
| Feedback | Providing possibilities for the audience to seek clarification on the relayed information. |

**Step 2: Designing the fuzzy linguistic scale**

The fuzzy linguistic scale in Table A1.2 was used to collect feedback from the experts.

**Table A1.2. The Fuzzy Linguistic Scale for the Respondents’ Evaluations**

| **Linguistic terms** | **Triangular fuzzy numbers** |
| --- | --- |
| No influence (N) | (0.000, 0.000, 0.250) |
| Very low influence (VL) | (0.000, 0.250, 0.500) |
| Low influence (L) | (0.250, 0.500, 0.750) |
| High influence (H) | (0.500, 0.750, 1.000) |
| Very high influence (VH) | (0.750, 1.000, 1.000) |

**Step 3: Computing the initial direct relation matrix**

The combined initial direct relation fuzzy matrix for all respondents is indicated in Table A1.3 below. It was obtained by computing the average of the initial direct relation matrices for individual respondents, as per Equation 2 was used to compute the initial direct relation fuzzy matrix.

**Table A1.3. Initial Direct Relation Fuzzy Matrix**

|  | **AM1** | **AM2** | **AM3** | **AM4** | **AM5** | **AM6** | **AM7** | **AM8** | **AM9** | **AM10** | **AM11** | **AM12** | **AM1** | **AM2** | **AM3** | **AM4** | **AM5** | **AM6** | **AM7** | **AM8** | **AM9** | **AM10** | **AM11** | **AM12** | **AM1** | **AM2** | **AM3** | **AM4** | **AM5** | **AM6** | **AM7** | **AM8** | **AM9** | **AM10** | **AM11** | **AM12** |
| --- | --- | --- | --- | --- | --- | --- | --- | --- | --- | --- | --- | --- | --- | --- | --- | --- | --- | --- | --- | --- | --- | --- | --- | --- | --- | --- | --- | --- | --- | --- | --- | --- | --- | --- | --- | --- |
|  | **K’ij** | | | | | | | | | | | | **L’ij** | | | | | | | | | | | | **M’ij** | | | | | | | | | | | |
| **AM1** | 0.000 | 0.219 | 0.219 | 0.266 | 0.203 | 0.266 | 0.250 | 0.422 | 0.281 | 0.422 | 0.438 | 0.438 | 0.000 | 0.469 | 0.469 | 0.453 | 0.422 | 0.453 | 0.500 | 0.672 | 0.531 | 0.672 | 0.688 | 0.688 | 0.000 | 0.719 | 0.719 | 0.703 | 0.672 | 0.703 | 0.750 | 0.891 | 0.781 | 0.891 | 0.922 | 0.938 |
| **AM2** | 0.625 | 0.000 | 0.641 | 0.609 | 0.594 | 0.641 | 0.656 | 0.656 | 0.406 | 0.406 | 0.578 | 0.563 | 0.875 | 0.000 | 0.891 | 0.859 | 0.844 | 0.891 | 0.906 | 0.906 | 0.656 | 0.656 | 0.828 | 0.813 | 1.000 | 0.000 | 1.000 | 1.000 | 0.969 | 1.000 | 1.000 | 1.000 | 0.844 | 0.844 | 1.000 | 0.984 |
| **AM3** | 0.375 | 0.266 | 0.000 | 0.250 | 0.516 | 0.484 | 0.344 | 0.484 | 0.500 | 0.484 | 0.297 | 0.438 | 0.625 | 0.516 | 0.000 | 0.500 | 0.766 | 0.734 | 0.594 | 0.734 | 0.750 | 0.734 | 0.547 | 0.688 | 0.844 | 0.750 | 0.000 | 0.734 | 0.953 | 0.922 | 0.828 | 0.938 | 0.922 | 0.938 | 0.781 | 0.922 |
| **AM4** | 0.516 | 0.219 | 0.500 | 0.000 | 0.484 | 0.250 | 0.297 | 0.297 | 0.266 | 0.125 | 0.125 | 0.453 | 0.766 | 0.469 | 0.750 | 0.000 | 0.734 | 0.500 | 0.547 | 0.547 | 0.516 | 0.375 | 0.375 | 0.703 | 0.953 | 0.719 | 0.922 | 0.000 | 0.922 | 0.750 | 0.797 | 0.797 | 0.766 | 0.625 | 0.625 | 0.938 |
| **AM5** | 0.547 | 0.484 | 0.313 | 0.484 | 0.000 | 0.359 | 0.438 | 0.453 | 0.359 | 0.438 | 0.422 | 0.484 | 0.797 | 0.734 | 0.563 | 0.734 | 0.000 | 0.609 | 0.688 | 0.703 | 0.609 | 0.688 | 0.672 | 0.734 | 0.984 | 0.922 | 0.813 | 0.938 | 0.000 | 0.859 | 0.922 | 0.938 | 0.859 | 0.906 | 0.906 | 0.969 |
| **AM6** | 0.500 | 0.516 | 0.375 | 0.391 | 0.500 | 0.000 | 0.469 | 0.422 | 0.438 | 0.469 | 0.453 | 0.453 | 0.750 | 0.766 | 0.625 | 0.641 | 0.750 | 0.000 | 0.719 | 0.672 | 0.688 | 0.719 | 0.703 | 0.703 | 0.938 | 0.938 | 0.859 | 0.875 | 0.969 | 0.000 | 0.938 | 0.891 | 0.891 | 0.922 | 0.922 | 0.938 |
| **AM7** | 0.563 | 0.375 | 0.375 | 0.547 | 0.547 | 0.375 | 0.000 | 0.594 | 0.594 | 0.375 | 0.359 | 0.563 | 0.813 | 0.625 | 0.625 | 0.797 | 0.797 | 0.625 | 0.000 | 0.844 | 0.844 | 0.625 | 0.609 | 0.813 | 0.969 | 0.828 | 0.844 | 0.969 | 0.969 | 0.813 | 0.000 | 1.000 | 1.000 | 0.813 | 0.813 | 1.000 |
| **AM8** | 0.469 | 0.484 | 0.281 | 0.500 | 0.484 | 0.484 | 0.281 | 0.000 | 0.313 | 0.297 | 0.281 | 0.516 | 0.719 | 0.734 | 0.531 | 0.750 | 0.734 | 0.734 | 0.531 | 0.000 | 0.563 | 0.547 | 0.531 | 0.750 | 0.953 | 0.953 | 0.781 | 0.984 | 0.969 | 0.969 | 0.781 | 0.000 | 0.813 | 0.797 | 0.781 | 0.938 |
| **AM9** | 0.422 | 0.281 | 0.344 | 0.328 | 0.328 | 0.281 | 0.281 | 0.469 | 0.000 | 0.266 | 0.250 | 0.469 | 0.672 | 0.531 | 0.594 | 0.578 | 0.578 | 0.531 | 0.531 | 0.719 | 0.000 | 0.516 | 0.469 | 0.719 | 0.906 | 0.781 | 0.844 | 0.828 | 0.828 | 0.781 | 0.781 | 0.953 | 0.000 | 0.766 | 0.719 | 0.969 |
| **AM10** | 0.547 | 0.594 | 0.594 | 0.641 | 0.531 | 0.547 | 0.625 | 0.516 | 0.531 | 0.000 | 0.516 | 0.547 | 0.797 | 0.844 | 0.844 | 0.891 | 0.781 | 0.797 | 0.875 | 0.766 | 0.781 | 0.000 | 0.766 | 0.797 | 0.969 | 0.984 | 1.000 | 1.000 | 0.969 | 0.969 | 1.000 | 0.969 | 0.969 | 0.000 | 0.969 | 0.969 |
| **AM11** | 0.547 | 0.547 | 0.609 | 0.563 | 0.547 | 0.609 | 0.531 | 0.531 | 0.531 | 0.531 | 0.000 | 0.578 | 0.797 | 0.797 | 0.859 | 0.813 | 0.797 | 0.859 | 0.781 | 0.781 | 0.781 | 0.781 | 0.000 | 0.828 | 0.969 | 0.984 | 1.000 | 1.000 | 1.000 | 1.000 | 1.000 | 1.000 | 1.000 | 1.000 | 0.000 | 1.000 |
| **AM12** | 0.297 | 0.234 | 0.266 | 0.266 | 0.250 | 0.281 | 0.250 | 0.250 | 0.281 | 0.281 | 0.328 | 0.000 | 0.531 | 0.469 | 0.500 | 0.500 | 0.500 | 0.516 | 0.500 | 0.500 | 0.516 | 0.531 | 0.578 | 0.000 | 0.781 | 0.719 | 0.750 | 0.750 | 0.750 | 0.766 | 0.750 | 0.750 | 0.766 | 0.781 | 0.828 | 0.000 |

**Note:** A1=Naming, A2=Emotional anchoring, A3=Thematic anchoring, A4=Use of antinomies, A5=Use of metaphors, A6=Understanding the cognitive ability of the audience, A7=Crafting engaging messages, A8=Timing, A9=Cultural Consideration, A10=Consistency, A11=Feedback.

**Step 4: Normalizing the direct relation fuzzy matrix**

The normalized direct relation matrix was normalized by dividing each of the elements of the initial direct relation matrix by the maximum value of the sums of the rows and columns of the initial direct relation matrix (Equation 4). Thus, the elements were divided by 26.141, which is the highest value obtained using Equation 3.

**Table A1.4. Normalized Direct Relation Fuzzy Matrix**

|  | **AM1** | **AM2** | **AM3** | **AM4** | **AM5** | **AM6** | **AM7** | **AM8** | **AM9** | **AM10** | **AM11** | **AM12** | **AM1** | **AM2** | **AM3** | **AM4** | **AM5** | **AM6** | **AM7** | **AM8** | **AM9** | **AM10** | **AM11** | **AM12** | **AM1** | **AM2** | **AM3** | **AM4** | **AM5** | **AM6** | **AM7** | **AM8** | **AM9** | **AM10** | **AM11** | **AM12** |
| --- | --- | --- | --- | --- | --- | --- | --- | --- | --- | --- | --- | --- | --- | --- | --- | --- | --- | --- | --- | --- | --- | --- | --- | --- | --- | --- | --- | --- | --- | --- | --- | --- | --- | --- | --- | --- |
|  | **K’ij** | | | | | | | | | | | | **L’ij** | | | | | | | | | | | | **M’ij** | | | | | | | | | | | |
| **AM1** | 0.000 | 0.008 | 0.008 | 0.010 | 0.008 | 0.010 | 0.010 | 0.016 | 0.011 | 0.016 | 0.017 | 0.017 | 0.000 | 0.018 | 0.018 | 0.017 | 0.016 | 0.017 | 0.019 | 0.026 | 0.020 | 0.026 | 0.026 | 0.026 | 0.000 | 0.027 | 0.027 | 0.027 | 0.026 | 0.027 | 0.029 | 0.034 | 0.030 | 0.034 | 0.035 | 0.036 |
| **AM2** | 0.024 | 0.000 | 0.025 | 0.023 | 0.023 | 0.025 | 0.025 | 0.025 | 0.016 | 0.016 | 0.022 | 0.022 | 0.033 | 0.000 | 0.034 | 0.033 | 0.032 | 0.034 | 0.035 | 0.035 | 0.025 | 0.025 | 0.032 | 0.031 | 0.038 | 0.000 | 0.038 | 0.038 | 0.037 | 0.038 | 0.038 | 0.038 | 0.032 | 0.032 | 0.038 | 0.038 |
| **AM3** | 0.014 | 0.010 | 0.000 | 0.010 | 0.020 | 0.019 | 0.013 | 0.019 | 0.019 | 0.019 | 0.011 | 0.017 | 0.024 | 0.020 | 0.000 | 0.019 | 0.029 | 0.028 | 0.023 | 0.028 | 0.029 | 0.028 | 0.021 | 0.026 | 0.032 | 0.029 | 0.000 | 0.028 | 0.036 | 0.035 | 0.032 | 0.036 | 0.035 | 0.036 | 0.030 | 0.035 |
| **AM4** | 0.020 | 0.008 | 0.019 | 0.000 | 0.019 | 0.010 | 0.011 | 0.011 | 0.010 | 0.005 | 0.005 | 0.017 | 0.029 | 0.018 | 0.029 | 0.000 | 0.028 | 0.019 | 0.021 | 0.021 | 0.020 | 0.014 | 0.014 | 0.027 | 0.036 | 0.027 | 0.035 | 0.000 | 0.035 | 0.029 | 0.030 | 0.030 | 0.029 | 0.024 | 0.024 | 0.036 |
| **AM5** | 0.021 | 0.019 | 0.012 | 0.019 | 0.000 | 0.014 | 0.017 | 0.017 | 0.014 | 0.017 | 0.016 | 0.019 | 0.030 | 0.028 | 0.022 | 0.028 | 0.000 | 0.023 | 0.026 | 0.027 | 0.023 | 0.026 | 0.026 | 0.028 | 0.038 | 0.035 | 0.031 | 0.036 | 0.000 | 0.033 | 0.035 | 0.036 | 0.033 | 0.035 | 0.035 | 0.037 |
| **AM6** | 0.019 | 0.020 | 0.014 | 0.015 | 0.019 | 0.000 | 0.018 | 0.016 | 0.017 | 0.018 | 0.017 | 0.017 | 0.029 | 0.029 | 0.024 | 0.025 | 0.029 | 0.000 | 0.027 | 0.026 | 0.026 | 0.027 | 0.027 | 0.027 | 0.036 | 0.036 | 0.033 | 0.033 | 0.037 | 0.000 | 0.036 | 0.034 | 0.034 | 0.035 | 0.035 | 0.036 |
| **AM7** | 0.022 | 0.014 | 0.014 | 0.021 | 0.021 | 0.014 | 0.000 | 0.023 | 0.023 | 0.014 | 0.014 | 0.022 | 0.031 | 0.024 | 0.024 | 0.030 | 0.030 | 0.024 | 0.000 | 0.032 | 0.032 | 0.024 | 0.023 | 0.031 | 0.037 | 0.032 | 0.032 | 0.037 | 0.037 | 0.031 | 0.000 | 0.038 | 0.038 | 0.031 | 0.031 | 0.038 |
| **AM8** | 0.018 | 0.019 | 0.011 | 0.019 | 0.019 | 0.019 | 0.011 | 0.000 | 0.012 | 0.011 | 0.011 | 0.020 | 0.027 | 0.028 | 0.020 | 0.029 | 0.028 | 0.028 | 0.020 | 0.000 | 0.022 | 0.021 | 0.020 | 0.029 | 0.036 | 0.036 | 0.030 | 0.038 | 0.037 | 0.037 | 0.030 | 0.000 | 0.031 | 0.030 | 0.030 | 0.036 |
| **AM9** | 0.016 | 0.011 | 0.013 | 0.013 | 0.013 | 0.011 | 0.011 | 0.018 | 0.000 | 0.010 | 0.010 | 0.018 | 0.026 | 0.020 | 0.023 | 0.022 | 0.022 | 0.020 | 0.020 | 0.027 | 0.000 | 0.020 | 0.018 | 0.027 | 0.035 | 0.030 | 0.032 | 0.032 | 0.032 | 0.030 | 0.030 | 0.036 | 0.000 | 0.029 | 0.027 | 0.037 |
| **AM10** | 0.021 | 0.023 | 0.023 | 0.025 | 0.020 | 0.021 | 0.024 | 0.020 | 0.020 | 0.000 | 0.020 | 0.021 | 0.030 | 0.032 | 0.032 | 0.034 | 0.030 | 0.030 | 0.033 | 0.029 | 0.030 | 0.000 | 0.029 | 0.030 | 0.037 | 0.038 | 0.038 | 0.038 | 0.037 | 0.037 | 0.038 | 0.037 | 0.037 | 0.000 | 0.037 | 0.037 |
| **AM11** | 0.021 | 0.021 | 0.023 | 0.022 | 0.021 | 0.023 | 0.020 | 0.020 | 0.020 | 0.020 | 0.000 | 0.022 | 0.030 | 0.030 | 0.033 | 0.031 | 0.030 | 0.033 | 0.030 | 0.030 | 0.030 | 0.030 | 0.000 | 0.032 | 0.037 | 0.038 | 0.038 | 0.038 | 0.038 | 0.038 | 0.038 | 0.038 | 0.038 | 0.038 | 0.000 | 0.038 |
| **AM12** | 0.011 | 0.009 | 0.010 | 0.010 | 0.010 | 0.011 | 0.010 | 0.010 | 0.011 | 0.011 | 0.013 | 0.000 | 0.020 | 0.018 | 0.019 | 0.019 | 0.019 | 0.020 | 0.019 | 0.019 | 0.020 | 0.020 | 0.022 | 0.000 | 0.030 | 0.027 | 0.029 | 0.029 | 0.029 | 0.029 | 0.029 | 0.029 | 0.029 | 0.030 | 0.032 | 0.000 |

**Note:** A1=Naming, A2=Emotional anchoring, A3=Thematic anchoring, A4=Use of antinomies, A5=Use of metaphors, A6=Understanding the cognitive ability of the audience, A7=Crafting engaging messages, A8=Timing, A9=Cultural Consideration, A10=Consistency, A11=Feedback.

**Step 5: Obtaining the total relation fuzzy matrix**

The total relation fuzzy matrix was obtained by multiplying the normalized direct relation matrix by the inverse of the difference between the identity matrix and the normalized direct relation matrix for each level L, M and U, as per equations 5, 6 and 7.

**Table A1.5. The Total Relation Fuzzy Matrix**

|  | **AM1** | **AM2** | **AM3** | **AM4** | **AM5** | **AM6** | **AM7** | **AM8** | **AM9** | **AM10** | **AM11** | **AM12** | **AM1** | **AM2** | **AM3** | **AM4** | **AM5** | **AM6** | **AM7** | **AM8** | **AM9** | **AM10** | **AM11** | **AM12** | **AM1** | **AM2** | **AM3** | **AM4** | **AM5** | **AM6** | **AM7** | **AM8** | **AM9** | **AM10** | **AM11** | **AM12** |
| --- | --- | --- | --- | --- | --- | --- | --- | --- | --- | --- | --- | --- | --- | --- | --- | --- | --- | --- | --- | --- | --- | --- | --- | --- | --- | --- | --- | --- | --- | --- | --- | --- | --- | --- | --- | --- |
|  | **L’ij** | | | | | | | | | | | | **M’ij** | | | | | | | | | | | | **U’ij** | | | | | | | | | | | |
| **AM1** | 0.003 | 0.011 | 0.011 | 0.013 | 0.010 | 0.013 | 0.012 | 0.019 | 0.013 | 0.018 | 0.019 | 0.019 | 0.009 | 0.025 | 0.026 | 0.025 | 0.024 | 0.025 | 0.027 | 0.034 | 0.028 | 0.033 | 0.033 | 0.035 | 0.018 | 0.043 | 0.044 | 0.044 | 0.043 | 0.043 | 0.045 | 0.051 | 0.046 | 0.050 | 0.051 | 0.053 |
| **AM2** | 0.029 | 0.004 | 0.028 | 0.028 | 0.027 | 0.029 | 0.029 | 0.030 | 0.020 | 0.019 | 0.026 | 0.027 | 0.046 | 0.011 | 0.045 | 0.044 | 0.044 | 0.045 | 0.045 | 0.046 | 0.036 | 0.036 | 0.042 | 0.044 | 0.059 | 0.020 | 0.058 | 0.058 | 0.057 | 0.058 | 0.058 | 0.059 | 0.052 | 0.051 | 0.057 | 0.059 |
| **AM3** | 0.018 | 0.013 | 0.003 | 0.013 | 0.023 | 0.021 | 0.016 | 0.022 | 0.022 | 0.021 | 0.014 | 0.020 | 0.034 | 0.028 | 0.009 | 0.029 | 0.038 | 0.037 | 0.031 | 0.037 | 0.037 | 0.036 | 0.029 | 0.036 | 0.051 | 0.046 | 0.019 | 0.046 | 0.055 | 0.053 | 0.049 | 0.054 | 0.053 | 0.053 | 0.047 | 0.055 |
| **AM4** | 0.022 | 0.010 | 0.021 | 0.002 | 0.021 | 0.012 | 0.013 | 0.014 | 0.012 | 0.007 | 0.007 | 0.020 | 0.038 | 0.025 | 0.036 | 0.008 | 0.036 | 0.027 | 0.028 | 0.029 | 0.027 | 0.022 | 0.022 | 0.035 | 0.054 | 0.043 | 0.051 | 0.017 | 0.052 | 0.045 | 0.047 | 0.048 | 0.046 | 0.040 | 0.040 | 0.054 |
| **AM5** | 0.025 | 0.021 | 0.015 | 0.022 | 0.004 | 0.017 | 0.020 | 0.021 | 0.017 | 0.020 | 0.019 | 0.022 | 0.041 | 0.037 | 0.031 | 0.038 | 0.010 | 0.033 | 0.035 | 0.037 | 0.033 | 0.035 | 0.034 | 0.038 | 0.057 | 0.053 | 0.050 | 0.055 | 0.020 | 0.051 | 0.054 | 0.055 | 0.051 | 0.053 | 0.053 | 0.057 |
| **AM6** | 0.023 | 0.023 | 0.018 | 0.019 | 0.023 | 0.004 | 0.021 | 0.020 | 0.020 | 0.021 | 0.020 | 0.021 | 0.039 | 0.038 | 0.033 | 0.034 | 0.039 | 0.010 | 0.037 | 0.036 | 0.036 | 0.036 | 0.036 | 0.038 | 0.056 | 0.054 | 0.051 | 0.053 | 0.056 | 0.020 | 0.054 | 0.054 | 0.053 | 0.053 | 0.053 | 0.056 |
| **AM7** | 0.025 | 0.018 | 0.018 | 0.024 | 0.024 | 0.018 | 0.003 | 0.026 | 0.026 | 0.017 | 0.017 | 0.026 | 0.042 | 0.033 | 0.034 | 0.040 | 0.041 | 0.034 | 0.010 | 0.042 | 0.042 | 0.033 | 0.032 | 0.042 | 0.057 | 0.050 | 0.051 | 0.056 | 0.056 | 0.050 | 0.019 | 0.057 | 0.056 | 0.049 | 0.049 | 0.058 |
| **AM8** | 0.021 | 0.021 | 0.014 | 0.022 | 0.022 | 0.021 | 0.014 | 0.003 | 0.015 | 0.014 | 0.013 | 0.023 | 0.037 | 0.036 | 0.029 | 0.037 | 0.037 | 0.037 | 0.029 | 0.010 | 0.030 | 0.029 | 0.029 | 0.038 | 0.056 | 0.054 | 0.048 | 0.056 | 0.055 | 0.055 | 0.048 | 0.020 | 0.049 | 0.048 | 0.047 | 0.056 |
| **AM9** | 0.019 | 0.013 | 0.016 | 0.015 | 0.015 | 0.013 | 0.013 | 0.021 | 0.003 | 0.012 | 0.012 | 0.021 | 0.034 | 0.028 | 0.030 | 0.030 | 0.030 | 0.028 | 0.028 | 0.036 | 0.008 | 0.027 | 0.025 | 0.036 | 0.053 | 0.046 | 0.049 | 0.049 | 0.049 | 0.047 | 0.047 | 0.054 | 0.018 | 0.046 | 0.044 | 0.055 |
| **AM10** | 0.026 | 0.026 | 0.027 | 0.029 | 0.025 | 0.025 | 0.028 | 0.024 | 0.024 | 0.004 | 0.023 | 0.026 | 0.043 | 0.042 | 0.043 | 0.045 | 0.041 | 0.041 | 0.044 | 0.041 | 0.041 | 0.011 | 0.039 | 0.043 | 0.058 | 0.057 | 0.058 | 0.058 | 0.058 | 0.057 | 0.058 | 0.058 | 0.057 | 0.020 | 0.056 | 0.059 |
| **AM11** | 0.026 | 0.025 | 0.027 | 0.026 | 0.025 | 0.027 | 0.024 | 0.025 | 0.024 | 0.024 | 0.004 | 0.027 | 0.043 | 0.041 | 0.043 | 0.042 | 0.042 | 0.043 | 0.040 | 0.041 | 0.041 | 0.040 | 0.011 | 0.044 | 0.059 | 0.057 | 0.058 | 0.059 | 0.059 | 0.058 | 0.058 | 0.059 | 0.058 | 0.058 | 0.021 | 0.060 |
| **AM12** | 0.014 | 0.011 | 0.012 | 0.012 | 0.012 | 0.013 | 0.012 | 0.012 | 0.013 | 0.013 | 0.014 | 0.003 | 0.028 | 0.025 | 0.026 | 0.026 | 0.027 | 0.027 | 0.026 | 0.027 | 0.027 | 0.027 | 0.029 | 0.008 | 0.047 | 0.043 | 0.044 | 0.045 | 0.045 | 0.045 | 0.044 | 0.045 | 0.045 | 0.045 | 0.047 | 0.018 |

**Note:** A1=Naming, A2=Emotional anchoring, A3=Thematic anchoring, A4=Use of antinomies, A5=Use of metaphors, A6=Understanding the cognitive ability of the audience, A7=Crafting engaging messages, A8=Timing, A9=Cultural Consideration, A10=Consistency, A11=Feedback.

**Step 6: Defuzzifying the total relation fuzzy matrix**

To de-fuzzify the total relation matrix, the best non-fuzzy performance (BNP) method (Equation 9) was used. For all triangular fuzzy numbers of each factor, the L element was subtracted from the M element. This difference was then subtracted from the difference of the U element and the L element. The difference obtained was divided by 3, and then the quotient was summed with the L element.

**Table A1.6. The De-fuzzified Total Relation Matrix**

|  | **AM1** | **AM2** | **AM3** | **AM4** | **AM5** | **AM6** | **AM7** | **AM8** | **AM9** | **AM10** | **AM11** | **AM12** |
| --- | --- | --- | --- | --- | --- | --- | --- | --- | --- | --- | --- | --- |
| **AM1** | 0.006 | 0.017 | 0.017 | 0.019 | 0.017 | 0.019 | 0.018 | 0.024 | 0.019 | 0.024 | 0.025 | 0.026 |
| **AM2** | 0.033 | 0.007 | 0.033 | 0.032 | 0.032 | 0.033 | 0.033 | 0.034 | 0.025 | 0.025 | 0.031 | 0.032 |
| **AM3** | 0.024 | 0.019 | 0.006 | 0.019 | 0.028 | 0.027 | 0.022 | 0.027 | 0.027 | 0.027 | 0.020 | 0.026 |
| **AM4** | 0.028 | 0.017 | 0.026 | 0.006 | 0.026 | 0.018 | 0.020 | 0.020 | 0.019 | 0.013 | 0.013 | 0.026 |
| **AM5** | 0.030 | 0.027 | 0.021 | 0.028 | 0.007 | 0.023 | 0.026 | 0.027 | 0.023 | 0.025 | 0.025 | 0.029 |
| **AM6** | 0.029 | 0.028 | 0.024 | 0.025 | 0.029 | 0.007 | 0.027 | 0.026 | 0.026 | 0.027 | 0.026 | 0.028 |
| **AM7** | 0.030 | 0.023 | 0.023 | 0.030 | 0.030 | 0.023 | 0.007 | 0.031 | 0.031 | 0.023 | 0.022 | 0.031 |
| **AM8** | 0.027 | 0.027 | 0.020 | 0.028 | 0.028 | 0.027 | 0.020 | 0.007 | 0.021 | 0.020 | 0.020 | 0.029 |
| **AM9** | 0.025 | 0.019 | 0.022 | 0.021 | 0.022 | 0.019 | 0.019 | 0.027 | 0.006 | 0.019 | 0.018 | 0.027 |
| **AM10** | 0.031 | 0.031 | 0.032 | 0.033 | 0.030 | 0.030 | 0.032 | 0.030 | 0.030 | 0.007 | 0.029 | 0.031 |
| **AM11** | 0.031 | 0.030 | 0.032 | 0.031 | 0.031 | 0.032 | 0.030 | 0.031 | 0.030 | 0.030 | 0.007 | 0.032 |
| **AM12** | 0.020 | 0.017 | 0.018 | 0.018 | 0.018 | 0.019 | 0.018 | 0.018 | 0.019 | 0.019 | 0.020 | 0.006 |

**Note:** A1=Naming, A2=Emotional anchoring, A3=Thematic anchoring, A4=Use of antinomies, A5=Use of metaphors, A6=Understanding the cognitive ability of the audience, A7=Crafting engaging messages, A8=Timing, A9=Cultural Consideration, A10=Consistency, A11=Feedback.

**Step 7: Establishing and analyzing the F-DEMATEL diagram**

To obtain the f-DEMATEL causal diagram, the sums of the rows (D_i_) and sums of columns (R_i_) were obtained. Thereafter, the Di and Ri values for every factor were summed to obtain prominence values; and subtracted to obtain net effect values. The net effect values were used to categorize the factors as causal or effect factors. Factors with positive net values were categorized as causal factors, and those with negative net values were categorized as effect factors. In addition, a threshold value was set to determine whether the causal/effect factors were significant. The threshold value was obtained by summing the mean and standard deviation of the de-fuzzified total relation matrix, as recommended by (69). The threshold value established by this study was 0.031. Factors whose absolute net effect values were greater than the threshold were categorized as significant causal or effect factors. The results are indicated in Table 3 in the main text.
