# Supplementary material for: Exploring determinants of formation of cognitive anchors from altruistic messages: A fuzzy DEMATEL approach
Source: PLoS One. 2023 Nov 6;18(11):e0293841. doi: 10.1371/journal.pone.0293841 (PMC10627445; doi:10.1371/journal.pone.0293841)
Supplement: Attachment — Submitted filename: Response to Reviewers.docx [file pone.0293841.s002.docx]

Dear Reviewer,

The time and effort devoted to reviewing the manuscript are greatly appreciated. The insightful comments and valuable suggestions have significantly enhanced the paper's quality. In response to this feedback, all raised points have been thoroughly addressed, and the revisions have been meticulously highlighted within the manuscript. Below, there is a detailed point-by-point response to these comments and concerns to aid in the review process.

Reviewer #1: Overall, I think this article is innovative, but as far as the writing of the article is concerned, the language is not concise enough and there is a lot of room for improvement, and I think the article needs to be revised in some way.

1. The article does not give the fuzzy language scale and its corresponding values.

*Thank you for your suggestions. We have added a table showing the fuzzy language scale and its corresponding values (Table 1).*

2. This article is not very strict in the selection of factors, and although the article is a literature review for factor extraction, the factors still need to be screened to some extent so as to ensure the accuracy of the analysis results. For example, analysis of variance (ANOVA), etc.

*Thank you for your comment. We conducted Delphi surveys with the participants before using the factors in the final survey. We initially did not include an explanation for the Delphi survey due to space limitations. The information about the Delphi survey has since been added to the first paragraph of section ‘3.3 Procedure’. We feel it would also be important to add another study, say a survey, to enhance the robustness of the findings of the F-DEMATEL study. However, due to time constraints, we could not conduct another study. This is indicated as one of the limitations of the current study in section 6.*

3. The description of the factors is not sufficiently concise and the language should be more streamlined and a table format is recommended.

*Thank you for your recommendation. We enhanced the description of the factors to ensure that the link between every factor and anchoring is clear. We have also added a table with descriptions for each factor in the A1 file.*

4. The article lacks a description of the existing research gaps as well as the significance of this study. It is suggested that a description of the current research gaps as well as the significance of the study be added to the introduction.

*Thank you for your suggestions. We added two research gaps in the third and fourth paragraphs of the introduction and added the theoretical and practical significance of the study in the final paragraph of the introduction.*

5. The article lacks a well-structured technology roadmap. A technology roadmap would give the reader a clearer understanding of the overall structure of the article.

*Thank you for your comment. The paper’s roadmap is in the final sentence of the final paragraph of the introduction section.*

6. The article is long in the conclusion section, and I think it could be more concise.

*Thank you for the suggestions. We trimmed the discussion section and merged some subsections under it to make it more concise.*

7. In the results section, I don't think it is necessary to repeat the formulae, thus leading to an increase in the length of the article; a good article should be written in simple language.

*Thank you for the comment. We deleted the detailed explanations of the results under the table, as these necessitated the use of the equations. They appeared to be repetitions of what was explained in the first paragraph, and so we deleted them.*

8. The literature review section of the article is too long; an article's literature review should be developed in terms of the current research status, research gaps, and the significance of this study. In addition, I think chapter 2.5 should be treated as a separate chapter, which would make the structure of the article clearer.

*Thank you for the suggestions. We shortened the literature review and revised it to ensure that it explains the current status of the research, the gaps, and the significance of this study. We turned section 2.5 to chapter 3.*

Dear Reviewer,

The time and effort devoted to reviewing the manuscript are greatly appreciated. The insightful comments and valuable suggestions have significantly enhanced the paper's quality. In response to this feedback, all raised points have been thoroughly addressed, and the revisions have been meticulously highlighted within the manuscript. Below, there is a detailed point-by-point response to these comments and concerns to aid in the review process.

1. The writing standard of the entire manuscript is not satisfactory. It needs a major overhaul and professional English proofreading.

*Thank you for your comments. We have revised the manuscript substantially and had professional proofreading done on it.*

2. The Abstract is not satisfactory. The background is not clear. The research gap is not mentioned. The result is not mentioned. The writing standard is not satisfactory.

*Thank you for the observation. We have revised the abstract and added all relevant information in the abstract.*

3. Citations in the manuscript are not uniform. You have used the number format in most cases but there are some citations in different formats like page 6 (De Leeuw et al., 2022); Page 9 (Fang, 2021). Moreover, page numbers are given in both the top right and bottom center.

*Thank you for the observation, we have revised all citations and page numbers to ensure that they are uniform.*

4. In Introduction, Research Objectives are missing. Moreover, the research gap you are trying to address is not clear. The research contribution is not elaborately written.

*Thank you for the comments. We added the research gaps and objectives in the introduction. We also revised our writing of the research contributions in the introduction and section 6.2.*

5. Background of the study is not adequately explained in the Introduction.

*Thank you for the observation. We revised the background to make it more comprehensible.*

6. More recent papers published in 2022 and 2023 in reputed journals like Elsevier or Plos should be discussed and cited. Especially, since it’s a MCDM paper submitted in Plos, cite other recently published Plos paper that has used various MCDM methods, especially the DEMATEL variants, to establish your paper's relevance to this journal. Try citing the following recent papers on F DEMATEL.
• Kuzu, A. C. (2023). Application of fuzzy DEMATEL approach in maritime transportation: A risk analysis of anchor loss. Ocean Engineering, 273, 113786.
• Çelik, M. T., & Arslankaya, S. (2023). Analysis of quality control criteria in an business with the fuzzy DEMATEL method: Glass business example. Journal of Engineering Research, 11(2), 100039.
• Siraj, M. T., Debnath, B., Kumar, A., Bari, A. M., Samadhiya, A., & Payel, S. B. (2023). Evaluating barriers to sustainable boiler operation in the apparel manufacturing industry: Implications for mitigating operational hazards in the emerging economies. Plos one, 18(4), e0284423.

*Thank you for the suggestions. We have cited these papers and five other PlosOne papers.*

7. Any specific systematic literature Review technique applied for factor identification? This is not mentioned or discussed anywhere. Moreover, what are the research protocols used (search strings, timeline of search, inclusive & exclusive criteria) for literature search?

*Thank you for the comment. We have elaborated the research protocol for the extraction of the factors in “Section 4.3 Procedure”.*

8. The methodological study framework of the study is missing. Provide it in a pictorial format.

*Thank you for the comment. We have added the framework as Figure 1.*

9. In section 3.2, you mentioned that “Figure 1 indicates the process framework for the m-delphi/F-DEMATEL techniques used in this study”. But I don’t find anything about Delphi here.

*Thank you for the suggestion. We have deleted the figure as per your recommendation in comment 13. We have discussed M-DELPHI as a preliminary technique for scrutinizing the factors prior to their usage in the study in Section 4.3.*

10. Correct all typos through out the paper, such as “delphi” (It’s a process name, should be “Delphi”). You have written both f and F DEMATEL. Which one is correct?

*Thank you for the observation. This has been done. F-DEMATEL has been maintained throughout the paper.*

11. Why Fuzzy DEMATEL is used in this study over other methods? What are the salient advantages of F-DEMATEL over other MCDM methods?

*Thank you for the questions. We have added the explanation for why we used F-DEMATEL in SECTION “4.2 DEMATEL”.*

12. In section 3.2.2, you mentioned that the fuzzy linguistic scale and its corresponding values are provided in Table 1. But Table 1 defines the Fuzzy total relation matrix. The linguistic scale is not found.

*Thank you for the observation. We have added the Table under the section (which is now named Section 3.2.2).*

13. What do you mean by the process framework of DEMATEL in Figure 1? I have not seen such figure in any DEMATEL study. Delete this figure.

*Thank you for the recommendation. We deleted the figure accordingly.*

14. The experts' profile is not given. Provide it.

*Thank you for the recomendation. We have added the respondents’ profile in Table 2 (Demographics).*

15. You have just written equations in methods. But their applications in your problem are not defined. You should mention the outcome of your results with regard to each of the equations.

*Thank you for the commemnts. We added an appendix, A1, in which are contained the applications of the equations in each step of the F-DEMATEL calculation process. We have added the detailed explanation in the additional file to make the file easier to read and to avoid hitting the word limit in the main file.*

16. Where is the reference of this F-DEMATEL approach? From which research article did you adopt the methodological approach? Since you didn’t invent F DEMATEL here, you have to cite the source paper here.

*Thank you for the observation. We have indicated the source of the F-DEMATEL procedure in the paper. We adopted it from the paper below:*

Liao, C. H. (2020). Evaluating the social marketing success criteria in health promotion: A F-DEMATEL approach. *International Journal of Environmental Research and Public Health*, *17*(17), 6317. <https://doi.org/10.3390/ijerph17176317>.

17. How did you collect the data? What sampling approach did you adopt for your research? There should be a discussion on the survey design.

*Thank you for the question. We have added the information in section 4.3.*

18. In the Results, you have mentioned “After that, the direct relation matrix was normalized using Equation 3 (Table 1).” But you have defined Table 1 as Fuzzy total relation matrix. There are so many irregularities like this throughout the paper.

*Thank you for the observation. This has been rectified. We have used accurate terminologies for all tables.*

19. You have mentioned that Table 2 defines Cause and Effect relationships. But, you haven’t even defined which one are in cause group and which one are in effect group in the table. You should do that.

*Thank you for the observation. This has been done accordingly.*

20. How you achieved the degree of significance as: (The degree of significance of the causal and effect factors was decided using a cut-off point determined by the average (α=0.033))? This is actually threshold value I think. You should mention how the threshold value is calculated and obtained.

*Thank you for the observation. We have indicated the procedure for calculating the threshold value in the A1 file. We summed the mean and standard deviation of the de-fuzzified total relation matrix, a technique used by a prior study (Siraj et al., 2023).*

21. Where is the causal relationship diagram? You have just drawn the clustered cause-effect group diagram in Figure 2. But the causal interrelations between the factors diagram is missing.

*Thank you for your comment. We have added the causal relationship diagram as Figure 3.*

22. The description of the result and discussion is not satisfactory. You need to elaborate them.

*Thank you for the comments. We enhanced the analysis of the results in the discussion section.*

23. The distinction of this study's findings with other relevant studies are missing at the end of the discussion section.

*Thank you for the observation. We elaborated on the links between our findings and the findings of prior studies in the discussion section (Section 6).*

24. The theoretical implication needs to incorporate how it contributes theoretically to the existing literature.

*Thank you for the comment. We have enhanced the theoretical implications to ensure that there is clarity on how the paper contributes theoretically to existing literature.*

25. Where is the main body of the conclusion??

*Thank you for the observation. We added a conclusion section (Section 9) to the manuscript accordingly.*

26. The overall writing is very poor.
If authors must improve the paper addressing all these comments.

Thank you for your comments and suggestions. We appreciate the time and effort you dedicated to providing us with valuable, concise feedback.
